# Trends in Plant Breeding and Genetics

**DOI:** 10.3390/ijms27198475

**Published:** 2026-09-23

**Authors:** Andrés J. Cortés

**Affiliations:** Facultad de Ciencias Agrarias—Departamento de Ciencias Forestales, Universidad Nacional de Colombia—Sede Medellín, Medellín 050034, Colombia; ancortesv@unal.edu.co

As global ecosystems face intensifying pressures associated with climate change [1], emerging pathogens [2], land and water limitations [3], and the urgent need to sustainably increase food production [4], the development of more efficient and resilient crops has become a major scientific and societal priority. Fortunately, plant breeding is entering an era of unprecedented transformation driven by the convergence of quantitative genetics [5], high-throughput genomics [6], genomic selection [7], functional biology [8], and gene editing [9]. In this context, the Special Issue “Plant Breeding and Genetics: New Findings and Perspectives” brings together a diverse collection of original research articles and reviews that collectively illustrate how modern breeding frameworks are evolving from solely phenotype-based selection routines [10] toward increasingly phenotype–genotype-integrated “big data”-driven predictive approaches [11], which are still dependent on the generation of classical breeding populations. The contributions compiled here span multiple dimensions, including genome-wide germplasm characterization [12], candidate gene discovery [13] and functional genomics [14], genomic prediction [15] and molecular breeding [16], and advanced genome engineering technologies [17]. Together, these studies demonstrate how the integration of classical breeding principles with modern omics technologies, bioinformatics, synthetic biology and gene editing is expanding the usable spectrum of genetic diversity [18], while accelerating the development of productive, climate-resilient and sustainable crop systems [19]. In this way, the Special Issue advocates for interdisciplinary integration across the fields of crop science, genomics, physiology, molecular biology, computational sciences and evolutionary genetics to bridge paramount research gaps in the development and deployment of advanced plant improvement approaches [20] capable of meeting, in a sustainable and resilient manner, growing global food security demands [21]. Once the diverse disciplines that orbit the plant breeding spectrum converge, a much-needed modern synthesis would emerge between the basic and applied plant science and crop improvement fields. The current compilation offers a first glimpse into this regard by providing conceptual frameworks and practical approaches capable of assisting ongoing efforts to secure food production, climate change adaptation, and sustainable agricultural practices.

## 1. Broadening the Breeding Genepool Through Germplasm Screening

The rapid expansion of genomic technologies is transforming how plant germplasm is conserved [22], shared [23], characterized [24], and integrated [25] into breeding pipelines [26]. As modern agriculture faces increasing environmental pressures and the genetic bottleneck associated with intensive cultivar standardization [27], the exploration [28], usage [29], and mobilization [30] of underutilized and regionally adapted germplasm has become a major priority for plant improvement programs.

The methods available for leveraging germplasm resources, such as high-throughput sequencing, genome-wide SNP discovery, and scalable marker platforms, now allow pre-breeders to dissect population stratification [31], trace evolutionary trajectories, identify adaptive introgression signatures, accelerate the deployment of valuable alleles from landraces, and utilize wild relatives and secondary diversification centers in breeding programs. In this regard, Cortés et al. [32] carried out the first low-coverage whole-genome sequencing (lcWGS) assessment of avocado (*Persea americana*) germplasm from Tanzania, an emerging secondary center of diversification for this globally important perennial fruit tree crop. The study analyzed 95 farmer-selected “plus trees” sampled across strong elevational and climatic gradients spanning the low- to mid-altitude Morogoro region (498–1001 m a.s.l.) and the Southern Highlands of Iringa, Mbeya, and Ruvuma (931–1715 m a.s.l.). Authors further integrated this data with 205 previously published race-representative avocado genomes for ancestry inference and population structure analyses [33]. The team demonstrated that Tanzanian avocados form a predominantly West Indian-derived genepool, with most Morogoro trees exhibiting >80% West Indian ancestry consistent with adaptation to warm, humid lowland tropical environments. In contrast, trees from the Southern Highlands displayed detectable West Indian × Guatemalan and West Indian × Mexican introgression signatures, suggesting that local environmental filtering and farmer-mediated selection may already be favoring alleles associated with adaptation to cooler and higher-altitude agroecosystems. Despite the absence of pure Guatemalan or Mexican accessions in the sampled populations, the presence of admixed genotypes indicates early-stage diversification processes driven by recurrent recombination, allogamy, and local selection pressures within East African production systems. The study further linked these genomic patterns with phenotypic diversity [34] previously documented in Tanzanian avocado populations [35], including buttery, watery, pastose, and granular fruit textures—quality traits relevant for breeding diversification beyond the globally dominant var. “Hass” cultivar [36]. From a methodological perspective, the work generated a high-confidence panel of 10,460 SNPs filtered from whole-genome resequencing data using stringent depth, allele frequency, and missing-data thresholds, creating a scalable genomic resource suitable for genome-wide association studies (GWASs), genomic prediction, marker-assisted introgression [37], and nursery-stage early selection of elite rootstocks [38]. The authors further emphasized that the complete absence of northwestern South American genomic ancestry in Tanzanian avocados creates opportunities for future introgressive breeding with the Andean–Caribbean germplasm, recently identified as a cryptic, deeply divergent avocado clade [39]. The work positions East Africa as a previously underrecognized secondary diversification center for avocado that may harbor adaptive allele combinations absent from American primary and commercial genepools.

Wang et al. [40] developed a similar, more targeted high-resolution SNP fingerprinting platform for eggplant based on Kompetitive Allele-Specific PCR (KASP) technology [41]. Using whole-genome resequencing of 49 representative accessions, the authors identified hundreds of high-quality SNPs and subsequently developed a reduced panel of highly informative markers capable of discriminating among 280 eggplant accessions, resolving population stratification, and defining representative core collections for conservation and breeding applications. The study illustrates how the integration of genomics, bioinformatics, and molecular marker-assisted selection can modernize germplasm management and accelerate cultivar identification with greater precision, reproducibility, and scalability than traditional SSR-based approaches. The establishment of compact SNP panels for varietal traceability [42] and diversity assessment [43] provides practical tools for breeding programs seeking to enhance genetic gain, maintain broad adaptive variation, and support the development of resilient crop varieties under increasingly variable climatic conditions.

Exploring alternative screening platforms, Shafiq et al. [44] combined high-throughput specific-locus-amplified fragment sequencing (SLAF-seq) [45] with detailed phenotypic and photosynthetic characterization to investigate the genetic diversity of 174 globally sourced white clover (*Trifolium repens* L.) accessions. The study generated an extensive genomic resource comprising over 2.9 million SNPs, from which 102,025 high-quality markers were retained to resolve population structure and genetic relationships. Genome-wide analyses revealed up to three partially overlapping genetic groups, while agronomic clusters spanned large-leaved, high-biomass genotypes, and highly stoloniferous accessions adapted for persistence under grazing. Substantial variation was detected not only in morphological traits but also partially in photosynthetic parameters related to carbon assimilation, chlorophyll content and photochemical efficiency, highlighting the broad adaptive potential present within global white clover germplasm. The strong concordance between phenotypic and molecular clustering demonstrated that the SNP dataset captures agronomically usable variation, providing a valuable foundation not only for germplasm conservation and molecular fingerprinting, but also for quantitative trait nucleotide (QTN) mapping via GWAS and subsequent marker-assisted breeding, illustrating how high-density genomic characterization can accelerate the identification and deployment of adaptive diversity in forage crops.

Lastly, Werghi et al. [26] offered a comprehensive review of genome-wide genotyping technologies for plant germplasm characterization and their applications in pre-breeding. The study synthesized the development and comparative utility of major genotyping platforms—including SNP arrays, genotyping-by-sequencing (GBS) [46], DArTseq [47], RAD-seq [48], whole-genome resequencing, and low-coverage sequencing approaches [49]—for the large-scale characterization of genetic diversity preserved in germplasm banks, landraces, crop wild relatives, and elite cultivars. The authors emphasized that dense genome-wide marker datasets now enable assessments beyond simple diversity, supporting population structure analyses, identification of redundant accessions, detection of loci under selection, GWAS mapping, genomic prediction, and marker-assisted introgression of adaptive alleles into breeding populations. The team further discussed how advances in sequencing throughput, bioinformatics, and statistical genetics are transforming entire germplasm collections from passive antique repositories into knowable and usable genomic resources capable of accelerating precision breeding and climate adaptation. The authors also pinpointed the challenges associated with managing large genomic datasets, standardizing phenotypic data integration, and ensuring interoperability among germplasm databases [50], all of which remain critical bottlenecks for translating genomic germplasm information into pre-breeding schemes [51]. Despite these shortcomings, high-density genotyping is redefining the conservation and utilization of plant genetic resources, providing the genomic bases necessary for the development of improved populations and varieties [52].

Altogether, the studies presented in this section demonstrate that the expanding capacity to characterize genome-wide diversity in germplasm collections is reshaping the conceptual and practical foundations of plant pre-breeding [53]. The merging of high-resolution genotyping, population genomics, crop evolutionary genetics, and scalable marker platforms [54] illustrates how previously underexplored germplasm reservoirs—including secondary diversification centers, farmer-selected landraces, and locally adapted populations—can be transformed into accessible genomic resources for crop improvement [55]. In other words, genomic-assisted germplasm screening is broadening the available breeding genepool across diverse crop systems while simultaneously redefining plant genetic resources as reachable reservoirs of cryptic diversity capable of supporting future food security [56].

Nonetheless, the identification of adaptive standing represents only the first step toward its effective deployment in pre-breeding pipelines [57]. Translating genomic variation into stable agronomic gains would benefit from a more mechanistic understanding of the genes, regulatory pathways, molecular networks, and pangenomic modifications governing stress tolerance, plasticity, and adaptation [58]. In this context, the transition from genome-wide germplasm screening toward functional genomics and candidate gene validation represents a natural next phase for precision plant improvement [8], enabling breeders to move beyond descriptive diversity screenings toward the more precise dissection and manipulation of the biological processes underlying plant phenotypes.

## 2. Candidate Gene Discovery and Validation

While genome-wide germplasm screening provides the raw evolutionary and adaptive diversity necessary for crop improvement [59], the conversion of this variation into deployable breeding strategies increasingly depends on identifying the specific genes, regulatory networks, and molecular pathways that underlie phenotypic variation and environmental resilience [60]. Recent advances in functional genomics, comparative transcriptomics, gene family characterization, and transgenic validation are now enabling researchers to move beyond descriptive diversity analyses, toward the mechanistic dissection of adaptive traits across both crop and non-model plant systems [61]. In this context, candidate gene discovery bridges large-scale genomic resources and precision breeding applications, allowing the direct incorporation of stress-responsive regulators, signaling pathways, and developmental control networks into breeding pipelines [62]. Furthermore, decreasing technology costs are now enabling the discovery of these mechanisms in non-model, underutilized, perennial, medicinal, forestry, and extremophile species naturally exposed to harsh environmental conditions, where evolutionary selection has already refined complex physiological responses to drought, heat, salinity, and developmental constraints [63] (see [64]).

At the interface of functional genomics and climate-resilient breeding, Zhu et al. [65] carried out a genome-wide characterization of the basic leucine zipper (bZIP) transcription factor family in *Bergenia purpurascens*, a high-altitude medicinal species naturally adapted to extreme alpine environments but increasingly threatened by global warming-induced heat stress. The authors identified 55 *BpbZIP* genes using transcriptome-wide analyses and classified them into 13 phylogenetic subfamilies, revealing conserved-motif architectures and evolutionary relationships with *Arabidopsis thaliana* bZIP proteins. RNA-seq and RT-qPCR analyses, under heat stress conditions, demonstrated that 32 *BpbZIP* genes were differentially expressed, with eight genes significantly upregulated and *BpbZIP37* showing the strongest induction following exposure to high temperatures. Functional validation through heterologous overexpression in *A. thaliana* demonstrated that *BpbZIP37* substantially enhanced thermotolerance under 45 °C heat stress, increasing seedling survival rates from 22.2% in wild-type plants to approximately 50% in transgenic lines, while also sustaining root growth and reducing leaf bleaching during post-stress recovery. The study showed that *BpbZIP37* improved heat tolerance by reducing reactive oxygen species accumulation and increasing antioxidant enzyme activities, particularly catalase and peroxidase, thereby maintaining cellular redox homeostasis under thermal stress. The work integrates comparative genomics, stress transcriptomics, and functional molecular validation to provide one of the first mechanistic frameworks explaining heat adaptation in *B. purpurascens*, a species previously studied mainly for its medicinal metabolites and cold tolerance. The contribution suggests how transcription factor networks regulating oxidative stress responses may be exploited in modern breeding pipelines to improve thermotolerance, adaptive resilience, and sustainable utilization of medicinal plant resources.

Meanwhile, harnessing embryo regeneration in conifers, Li et al. [66] functionally characterized the miR156–*SPL* signaling network during somatic embryogenesis in Japanese larch (*Larix kaempferi*), a commercially important conifer species with notoriously long breeding cycles and limited regeneration efficiency. The authors identified four conserved *LkMIR156* genes distributed across the larch genome and demonstrated that their mature miRNAs target multiple SQUAMOSA PROMOTER BINDING PROTEIN-LIKE (SPL) transcription factors involved in embryo maturation and developmental phase transitions. Detailed temporal expression analyses across embryogenic stages showed that precursor transcripts of *LkMIR156* accumulated predominantly during early embryogenesis (days 1–7), whereas mature LkmiR156 molecules peaked during cotyledonary embryo development (days 21–42), indicating dynamic post-transcriptional regulation throughout embryo differentiation and dormancy establishment. Secondary structure prediction confirmed highly stable stem–loop precursor conformations, while phylogenetic analyses revealed strong evolutionary conservation with miR156 homologs from both gymnosperm and angiosperm species. Functional validation through ectopic overexpression of *LkMIR156b1* in *A. thaliana* resulted in significant increases in rosette leaf number, leaf area, and delayed vegetative phase transition, thereby confirming the conserved developmental role of the miR156–*SPL* module across divergent plant lineages. Overall, the work provides insights into one of the major constraints limiting large-scale clonal propagation and genomic-assisted breeding in conifers: the poor understanding of molecular regulators controlling somatic embryo maturation and regeneration competence. After integrating developmental transcriptomics, miRNA biology, and functional genomics, the authors were able to establish a molecular framework for improving somatic embryogenesis and accelerating precision breeding in forest trees, illustrating how regulatory RNA networks can be incorporated into modern forestry improvement strategies aimed at enhancing propagation efficiency.

Likewise, from the forestry sector, Jin et al. [67] carried out a comprehensive genome-wide characterization of the *SNF1-related protein kinase 2* (*SnRK2*) gene family in *Populus euphratica*, a desert poplar species naturally adapted to hyper-arid and saline ecosystems. The authors identified 11 *PeSnRK2* genes distributed across seven chromosomes, most containing the canonical nine-exon/eight-intron structure characteristic of highly conserved ABA-responsive kinase families in higher plants. Phylogenetic analyses grouped these genes into three evolutionary subfamilies consistent with the *Arabidopsis SnRK2* classification system, while promoter analyses revealed abundant ABA-responsive (ABRE), drought-, light-, and hormone-associated cis-regulatory elements, highlighting their central role in abiotic stress signaling. Functional expression profiling under polyethylene glycol (PEG)-induced osmotic stress and exogenous abscisic acid (ABA) treatments demonstrated strong induction of *PeSnRK2.6*, a homolog sharing approximately 89% sequence identity with the *Arabidopsis* drought regulator *OST1/SnRK2.6*, a master controller of ABA-mediated stomatal closure. Subcellular localization assays further showed *PeSnRK2.6* activity in both the nucleus and cytoplasm, supporting its dual role in transcriptional regulation and intracellular stress signaling. The study links ABA-mediated kinase signaling with stomatal regulation and water-use efficiency, two complementary physiological processes underlying drought adaptation in perennial woody species. The work provides one of the first mechanistic frameworks describing *SnRK2*-mediated drought signaling in a desert tree species, expanding current understanding of stress adaptation beyond annual model crops. The work also demonstrates how conserved kinase signaling networks can be exploited in modern breeding roadmaps to improve drought resilience, water-use efficiency, and climate adaptation in forestry species and perennial crops facing increasingly severe water-limitation scenarios.

Also in desert poplar, Song et al. [68] carried out a genome-wide analysis of the *auxin response factor* (*ARF*) gene family in Salicaceae species and the functional characterization of *PeARF18* during heteromorphic leaf development in *P. euphratica*. The authors identified 115 *ARF* genes across four Salicaceae genomes, including 35 members in *P. euphratica*, and classified them into three major phylogenetic clades. Collinearity analyses revealed that segmental duplication events were the primary drivers of *ARF* gene family expansion in poplar species, while promoter analyses identified numerous cis-regulatory elements associated with auxin responsiveness, drought signaling, abscisic acid regulation, and light-mediated developmental control. Transcriptomic profiling across the characteristic heteromorphic leaf series of *P. euphratica*—ranging from linear and lanceolate juvenile leaves to ovate and broad-ovate adult forms—showed strong differential expression of several *PeARF* genes, with *PeARF18* exhibiting particularly high expression during transitions toward broad-ovate mature leaf morphologies associated with enhanced drought adaptation. Subcellular localization assays confirmed nuclear localization of *PeARF18*, consistent with its role as a transcriptional regulator within auxin signaling pathways controlling leaf polarity, cell expansion, and developmental phase transitions. These processes may underlie heterophylly in *P. euphratica*, which is a key adaptive mechanism that improves hydraulic regulation, transpiration control, and environmental plasticity in hyper-arid desert ecosystems. Overall, the work provides one of the most detailed molecular frameworks to date for understanding auxin-mediated developmental plasticity in perennial woody tree species. This contribution further demonstrates how regulatory gene networks governing morphological adaptation, historically regarded as highly polygenetic, can be incorporated into modern forestry pre-breeding and breeding pipelines, despite their long generation times, aimed at improving long-term resilience, water-use efficiency, and adaptation.

As illustrated in the examples above, modern functional genomics approaches—including transcriptome profiling, phylo-transcriptomics, regulatory RNA biology, promoter analyses, and inter-specific heterologous validation—are accelerating the discovery of key adaptive regulators while expanding the molecular toolkit available for improving thermotolerance, regeneration efficiency, developmental plasticity, drought resilience, and climate adaptation to current and future environmental conditions across diverse plant systems [69]. Altogether, this exquisite repertoire of studies also demonstrate that functional genomics is rapidly transforming plant breeding from a predominantly empirical discipline into a mechanistic and increasingly predictive science guided by a more detailed knowledge of the underlying genetic bases of key relevant trait variation. The contributions also reinforce how key adaptive regulators controlling thermotolerance, regeneration competence, developmental plasticity, and drought signaling can be identified and functionally deployed, despite polygenic architectures, across diverse medicinal, forestry, crop, and extremophile species [70]. This growing capacity to dissect the molecular architecture of complex adaptive traits provides the genetic foundation necessary for the next generation of precision breeding strategies [71]. However, identifying adaptive genes and regulatory pathways alone is insufficient for fully realizing their agronomic potential [72]. Their effective incorporation into breeding programs increasingly depends on the development of modern pre-breeding selection frameworks capable of rapidly converting genomic information into achievable genetic gain across variable environments [73]. In this context, transitioning from candidate gene discovery and validation toward gene-guided genomic prediction, marker-assisted pyramiding, and genomic selection models offers an opportunity step in accelerating climate-resilient crop improvement, enabling breeders to integrate the functional molecular bases into customized, scalable, “big data”-driven selection strategies [74].

## 3. Modernizing and Fast-Tracking Selection Proxies and Schemes

Identifying exotic germplasm and candidate genes underlying key traits is the foundation of a broader transformation currently underway in plant breeding. After all, modernization of selection schemes capable of efficiently converting genomic information into rapid and predictable genetic gain under increasingly unstable environmental conditions is a major need. Traditional breeding pipelines, often constrained by long generation intervals, extensive phenotyping requirements, and limited capacity to capture complex polygenic variation [75], are increasingly being complemented by genetic-enabled strategies integrating genome-wide marker screening platforms, marker-assisted pyramiding [76,77], and genomic selection models [78,79]. These approaches are now changing how breeders exploit and utilize genetic diversity, enabling earlier and more accurate selection proxies, accelerated introgression of exotic alleles from wild relatives, and simultaneous improvement of complex quantitative traits such as yield stability, disease resistance, and abiotic stress tolerance.

At this fascinating allelic deployment phase, López-Hernández et al. [80] aimed to leverage introgressive breeding through genomic prediction in interspecific hybrids between common bean (*Phaseolus vulgaris*) and the heat- and drought-adapted tepary bean (*P. acutifolius*) [81]. The authors used a panel of 87 advanced hybrid lines genotyped through GBS [82], retrieving 15,645 SNP markers, and implemented multiple Bayesian regression frameworks to predict agronomic performance across contrasting humid and dry environments in the Colombian Caribbean [83]. The study identified BayesC as the most accurate predictive model for yield components and biomass-related traits, achieving predictive abilities above 0.70 for key variables such as yield per plant, seed biomass, and pod number, thereby demonstrating the plausibility of genomic-enabled selection for climate adaptation in grain legumes. The authors also revealed that prediction accuracy was consistently higher when using the full SNP dataset compared to GWAS-derived marker subsets, highlighting the importance of capturing polygenic “missing heritability” in complex adaptive traits. The work offers a framework to accelerate breeding cycles, reduce phenotyping costs, and introgress adaptive alleles from the wild into elite lines.

Also in common bean, Benchimol-Reis et al. [84] synthesized the molecular breeding strategies for fungal resistance. The review examined eleven major fungal diseases affecting bean production—including anthracnose (*Colletotrichum lindemuthianum*), angular leaf spot (*Pseudocercospora griseola*), Fusarium wilt, white mold, charcoal rot and rust—and integrated current knowledge on resistance genes, quantitative resistance loci (QRLs) and pathogen-responsive defense pathways associated with each disease. The study also compiled chromosomal locations and functional effects of numerous resistance loci, including major-effect genes such as *Pm1* and *Pm2* for powdery mildew resistance, while also highlighting polygenic resistance architectures controlled through jasmonic acid, ethylene, salicylic acid and reactive oxygen species signaling networks. Beyond summarizing host–pathogen interactions, the review exemplified how modern genomic tools—including SNP arrays, KASP markers, GBS, GWAS, genomic selection, pan-genomics and CRISPR/Cas9-mediated gene editing—are transforming resistance breeding pipelines by enabling precise marker-assisted pyramiding of multiple resistance loci into elite cultivars. Finally, the authors further discussed how climate change is intensifying fungal disease pressure through altered temperature and humidity regimes, thereby increasing the urgency for durable and broad-spectrum resistance strategies capable of sustaining bean productivity under increasingly unpredictable environments. This contribution provides both a conceptual and practical framework for accelerating the development of climate-resilient bean cultivars with stable and long-lasting fungal resistance, illustrating the inevitable transition toward genomics-assisted crop protection and sustainable agricultural intensification.

Together, the studies highlighted in this section illustrate how genomic selection and molecular breeding strategies are fast-tracking crop improvement pipelines while reducing the costs, time, and uncertainty traditionally associated with breeding [85]. In parallel, modern genomics-assisted breeding frameworks are already contributing to the pressing need to improve crop sustainability while maintaining productivity across highly variable and unpredictable agroecological scenarios [86]. These modern selection frameworks are accelerating the transition from genomic discovery to practical crop improvement by integrating genome-wide marker platforms, resistance locus pyramiding, genomic prediction, and more refined genomic selection and eco-evolutionary models [87]. Such developments are making that complex adaptive traits—including yield stability, drought tolerance, and quantitative disease resistance—to be improved with unprecedented precision and efficiency across variable agroecological conditions [88]. All these advances also highlight a broader conceptual shift in plant breeding: from solely phenotype-driven selection toward increasingly genomic-enabled, “big data”-driven, probabilistic-assisted systems capable of capturing highly polygenic adaptive architectures and their environmental interactions [89,90,91]. Nevertheless, advanced genomic-assisted selection strategies remain dependent on the generation of breeding populations that span superior individuals, as well as on the capacity to manipulate, validate, and deploy targeted genetic variation within breeding pipelines. In this context, the next major frontier lies in overcoming the biological and technical bottlenecks limiting transformation, regeneration, and genome engineering across many economically important but tissue-culture recalcitrant species [92]. The incorporation of modern biotech—including optimized regeneration systems, synthetic biology platforms and programmable genome editing technologies—therefore represents an imminent natural and necessary extension of genomics-assisted breeding, enabling increasingly precise, rapid and design-oriented approaches for more customizable crops.

## 4. Turbocharging Plant Improvement

As indirect selection approaches such as genomic selection strategies continue to accelerate genetic gain, the next major frontier in plant improvement lies in overcoming the biological and technological bottlenecks that limit the direct implementation of advanced biotech approaches, specifically transgenesis and CRISPR/Cas9 gene editing [93]. Despite remarkable progress in genomics-assisted breeding, the practical deployment of genome editing, transgenic engineering, synthetic biology, and functional validation systems remains constrained by challenges such as low transformation efficiency, genotype-dependent regeneration capacity, complex polyploid genomes, and regulatory uncertainty surrounding emerging genomic technologies [94]. In this context, these resources are increasingly shifting from complementary breeding tools toward central platforms capable of dramatically shortening breeding cycles, expanding the accessible spectrum of genetic variation and enabling highly targeted manipulation of multiple desirable traits [95]. Advances in tissue culture optimization, transformation systems, synthetic reporter technologies and programmable genome editing are now opening new opportunities to directly engineer stress resilience, nutritional quality, pathogen resistance and developmental performance across both model and recalcitrant crop systems [96]. While these innovations are not only improving technical efficiency but also redefining the conceptual boundaries of plant breeding itself, moving the field toward more predictive, design-oriented, cost-effective, and increasingly polygenic genomic architectures is desired. In this sense, the studies highlighted below illustrate how modern technologies are turbocharging plant improvement pipelines by integrating regeneration biology, synthetic biology, transformation engineering, and genome editing into scalable strategies for accelerating food security, climate adaptation, and sustainable agricultural innovation.

Given the unavoidable importance of regeneration across multiple applications, Ionas et al. [97] addressed genotype-specific regeneration capacity in sunflower (*Helianthus annuus*), a crop that remains highly recalcitrant to tissue culture and genetic transformation despite its global agricultural importance. Evaluating 18 sunflower genotypes across contrasting regeneration media, the study combined temporal morphogenesis profiling, hierarchical clustering, and genotype-by-medium interaction analyses to identify distinct organogenic response patterns and develop predictive frameworks for regeneration efficiency. The authors classified genotypes into four regeneration multivariate clusters, including highly regenerative lines such as NO-SU-12 and AS-1-PR, intermediate responders, and completely non-regenerative genotypes such as AB-OR-8 and FE-7. Comparative analyses demonstrated that the R4 medium strongly enhanced organogenesis and root induction relative to the S1 medium, emphasizing the critical influence of hormonal composition on morphogenic competence. Temporal profiling revealed that early root formation at 14 days represented a robust predictive marker of subsequent regeneration success, whereas callus formation showed weak and inconsistent correlations with shoot and root development, challenging the conventional use of callogenesis as a proxy for regeneration potential. Complementary experiments manipulating sucrose concentrations in the model genotype HA-26-PR further demonstrated that although sucrose promoted callus proliferation, it failed to induce efficient organogenesis, highlighting the complex interaction between carbon signaling, osmotic regulation, and developmental differentiation pathways during in vitro regeneration. Because regeneration efficiency remains one of the principal barriers limiting the implementation of the revolutionary CRISPR/Cas genome editing and transgenic breeding in sunflower, the establishment of genotype-specific predictive regeneration protocols has major implications for accelerating functional genomics and precision breeding in this economically important oilseed crop. Overall, the work provides a scalable strategy for overcoming regeneration bottlenecks and improving transformation efficiency in genetically recalcitrant species, thereby strengthening the technological toolbox available for future climate-resilient crop improvement.

Also key for transformation success screening, He et al. [98] established an optimized RUBY reporter-assisted transformation system for banana (*Musa* spp.), coupled with the metabolic engineering of betalain biosynthesis for nutritional enhancement. The study employed *Agrobacterium*-mediated transformation of embryogenic cell suspensions using a construct containing the RUBY cassette, which integrates the coordinated expression of BvCYP76AD1, BvDODA1, and cDOPA5GT, three key genes controlling betalain biosynthesis. Transgenic events accumulated intense red-purple pigmentation in *calli*, regenerated plantlets, and fruit tissues, enabling rapid visual identification of successful transformants without the need for antibiotic-based or fluorescence screening systems. The optimized platform reduced transformation screening time from approximately 45 days to 15 days while maintaining stable transgene expression throughout plant development. Biochemical analyses confirmed substantial betalain accumulation in engineered fruits, reaching concentrations of 1.78 mg/g fresh weight, thereby demonstrating the feasibility of producing nutritionally enriched bananas with enhanced antioxidant properties. The work has addressed one of the major constraints in banana improvement, which is the low efficiency and lengthy selection procedures associated with genetic transformation in a crop characterized by sterility, polyploidy, and an extremely narrow genetic base vulnerable to devastating pathogens such as *Fusarium oxysporum* f. sp. *cubense* tropical race 4 (*Foc* TR4). Ultimately, integrating visible reporter technologies, synthetic biology, and transformation optimization provides a scalable framework for accelerating functional genomics, genome editing, and resistance breeding in banana.

Finally, Koller [99] reviewed the potential of new genomic techniques (NGTs) to overcome long-standing biological constraints in plant breeding. Focusing on CRISPR/Cas-based recombinant enzymatic mutagens (REMs), the work examined how targeted genome editing technologies can circumvent natural genomic stabilization mechanisms that traditionally limit the generation and fixation of novel genetic variation during conventional breeding. The author argued that, unlike more stochastic mutagenesis approaches based on radiation or chemical agents, modern NGTs actively interfere with endogenous DNA repair pathways, thereby enabling the creation of targeted allelic combinations and genomic configurations that would be unlikely to emerge solely through natural recombination or classical recurrent breeding. The work also placed these technological advances within an evolutionary and regulatory framework, discussing how increasingly sophisticated editing platforms—including base editing, prime editing, multiplex CRISPR systems, and AI-assisted genomic design—are expanding the scope and precision of crop improvement strategies. The study further addressed the regulatory implications of these technologies, emphasizing that current biosafety frameworks may insufficiently capture the mechanistic differences between conventional mutagenesis and highly directed genome engineering approaches. This contribution reflects the ongoing inevitable transition of plant breeding toward predictive, programmable, and highly targeted genomic manipulation frameworks capable of accelerating food security, resource-use efficiency, and climate adaptation under increasingly complex environmental and governmental scenarios.

The studies highlighted across this section reinforce how novel technologies are becoming the operational engine that transforms genomics, candidate gene discovery, and molecular breeding into gene-deployable crop improvement strategies. When viewed alongside the previous sections on germplasm screening, functional genomics and genomics-assisted selection, all thirteen contributions (Table 1) illustrate the emergence of an increasingly integrated plant breeding continuum spanning from the identification of adaptive diversity in underutilized germplasm, to the mechanistic dissection of stress-responsive regulatory networks, the predictive optimization of breeding decisions and ultimately the direct engineering of desirable phenotypes through advanced platforms [93]. However, overcoming persistent in vitro tissue culture regeneration bottlenecks, improving transformation efficiency, and expanding the precision and scalability of genome editing technologies are still key prerequisites for further realizing biotech full potential in modern breeding [100]. Together, these advances once again reinforce the broader conceptual transition in plant science—from observational and empirical selection-based improvement toward increasingly predictive, customizable, design-oriented, and system-level biological engineering frameworks capable of rapidly generating crops with enhanced productivity, nutritional quality, and resilience in the most vulnerable regions [101]. As climate uncertainty accentuates, and emerging pathogens and food insecurity intensify globally, the convergence of genomics, predictive breeding and gene editing technologies will likely define the future trajectory of sustainable and resilient plant improvement.

## 5. Perspectives

The studies compiled in this Special Issue illustrate that plant breeding is undergoing perhaps the most profound conceptual and technological transition in its history. What was once a largely phenotype-driven empirical discipline centered on incremental selection cycles is rapidly evolving into an integrative, predictive, and increasingly versatile field capable of dissecting, forecasting, and engineering complex traits across diverse crop systems [102]. From genome-wide germplasm characterization [103] and functional dissection of regulatory networks [104] to genomic prediction [105], molecular breeding [84] and gene editing [106], the contributions highlighted here demonstrate how the convergence of quantitative genetics, genomics, epigenetics, bioinformatics and synthetic biology is redefining the scale, timing and precision at which plant improvement can be performed [107]. Fortunately, these advances are concurrent with a time when global agriculture faces unprecedented challenges associated with climate extremes, emerging pathogens, resource scarcity and the urgent need to sustainably increase food production for a rapidly growing population [108].

One of the most prominent emerging trends across the studies contributed to this collection is the urgency of expanding the exploitable breeding germplasm beyond a historically narrow elite genepool [109]. The genomic characterization of secondary diversification centers, underutilized landraces, wild relatives, perennial species, and extremophile taxa demonstrates that a substantial reservoir of adaptive diversity remains largely untapped within global plant genetic resources [110]. Advances in lcWGS, GBS, high-density SNP screening platforms, and scalable marker systems are now making it possible to efficiently identify, trace, and mobilize adaptive alleles associated with drought tolerance, heat resilience, disease resistance, and environmental adaptation [111]. As climate change increasingly exposes the vulnerabilities shaped by decades of genetic homogenization in modern agriculture, the integration of these previously underexplored genomic resources into breeding pipelines will likely become one of the pivotal pillars of future crop resilience strategies.

At the same time, the studies in this Special Issue reinforce that the future of plant breeding will depend not only on broadening the genepool to identify standing adaptive diversity, but also on mechanistically understanding the molecular architectures underlying complex phenotypes [112]. Functional genomics approaches integrating transcriptomics, comparative genomics, phylogenetics, regulatory RNA biology, and transgenic validation are transforming candidate gene discovery into usable genic variation [113]. Many of the adaptive mechanisms described across the compiled studies were gathered in non-model systems—medicinal plants, forestry species, desert-adapted taxa, and perennial crops—thereby offering an expanded repertoire of novel stress-responsive regulators and developmental control networks. As multi-omics integration becomes more sophisticated, future breeding frameworks will likely move toward systems-level models capable of connecting genotype, regulatory architecture, physiology, and environmental response across multiple biological and geographic scales [114].

Equally transformative is the accelerating incorporation of predictive breeding frameworks into plant improvement programs [52]. Genome-wide marker platforms, genomic prediction, resistance locus pyramiding and probabilistic selection models are reshaping how breeders manage polygenic variation and selection uncertainty [115]. Rather than relying exclusively on lengthy phenotypic evaluations, pre-breeders can now increasingly forecast genetic performance early in breeding cycles, enabling faster introgression of adaptive alleles and more efficient improvement of complex quantitative architectures [116]. The growing integration of artificial intelligence, machine learning, environmental covariates, and high-throughput phenotyping with genomic prediction models is expected to further enhance predictive breeding systems (Figure 1).

While plant breeding is progressively transitioning from direct selection toward indirect genomic-enabled “big data”-driven decision-making frameworks, perhaps the most transformative frontier from this collection is the re-emerging expansion of biotech for plant improvement. Advances in regeneration biology, transformation engineering, synthetic biology, visible reporter systems, and programmable genome editing are dramatically expanding the range of species and traits subject to direct genetic manipulation. Nevertheless, the studies presented here also emphasize that major biological bottlenecks remain unresolved, particularly in recalcitrant plant species characterized by low transformation efficiency, genotype-dependent regeneration, sterility, polyploidy, or complex genome architectures. Overcoming these limitations will be essential to boost access to genome editing technologies across diverse agricultural systems, including major staple crops. Concurrently, the surging precision of CRISPR/Cas platforms [117], multiplex editing systems, base editing, prime editing, and AI-enabled genomic design contributes to the blurring of traditional boundaries separating breeding and synthetic biology, moving the field toward more customizable biological engineering frameworks [99].

Besides the impressive technological advances, this Special Issue also highlights the growing need for interdisciplinary integration across plant sciences. Future progress in forecasting plant improvement gains and outcomes [118] will depend on our ability to effectively merge population genomics, evolutionary ecology, quantitative genetics, molecular biology, crop physiology, computational biology, gene editing, and data science into unified predictive frameworks [119]. Integrating massive multidimensional datasets into interpretable and operationally deployable systems [120,121] must also be encouraged through a more diverse array of study systems, especially taking into account the absence of contributions on major staple crops to the current Special Issue compilation. This will require continued development of interoperable omics databases, scalable phenotyping infrastructures, advanced statistical methodologies, and open access computational resources capable of supporting global collaborative breeding efforts [122,123].

Last, but equally important, the accelerating pace of innovation in plant breeding also raises broader regulatory, ethical, and socioeconomic considerations. For instance, the emergence of increasingly precise genome editing technologies has challenged existing biosafety and regulatory frameworks originally designed around conventional transgenesis and random mutagenesis [96]. Similarly, equitable access to advanced breeding technologies remains uneven across regions and production systems, particularly in developing countries where climate vulnerability is often the greatest [124,125]. Ensuring that the benefits of such technologies reach smallholder farmers, underutilized opportunity and orphan crops, and regionally important agricultural systems will therefore represent a major priority for future agricultural sustainability and global food security [126].

## 6. Conclusions

The contributions compiled in the Special Issue “Plant Breeding and Genetics: New Findings and Perspectives” demonstrate that plant breeding is rapidly evolving toward an integrated, predictive, and more versatile discipline capable of addressing the unprecedented challenges imposed by climate change, emerging pathogens, and ultimately global food insecurity. Through the convergence of genome-wide germplasm characterization, genetic mapping, functional genomics, genomic prediction, and molecular breeding, the studies presented here illustrate how modern crop improvement is moving beyond traditional phenotype-based selection toward “big data”-driven and mechanistically informed predictive frameworks capable of accelerating the identification, forecasting, and deployment of genetic variation across diverse agricultural systems (Figure 2).

The collection not just highlights the expanding technical capacity to exploit underutilized germplasm, dissect complex regulatory networks and engineer desirable phenotypes with increasing precision, but also reinforces the growing need for interdisciplinary integration across quantitative genetics, genomics, physiology, computational biology and evolutionary biology. These fields together will continue redefining the conceptual boundaries of plant improvement, enabling more productive, sustainable and resilient crop systems. Ultimately, these foundational studies will help guide the next generation of breeding strategies aimed at securing food production, preserving genetic diversity and agro-ecosystem services, and strengthening global agricultural resilience under rapidly changing environmental conditions.

## Figures and Tables

**Figure 1:**
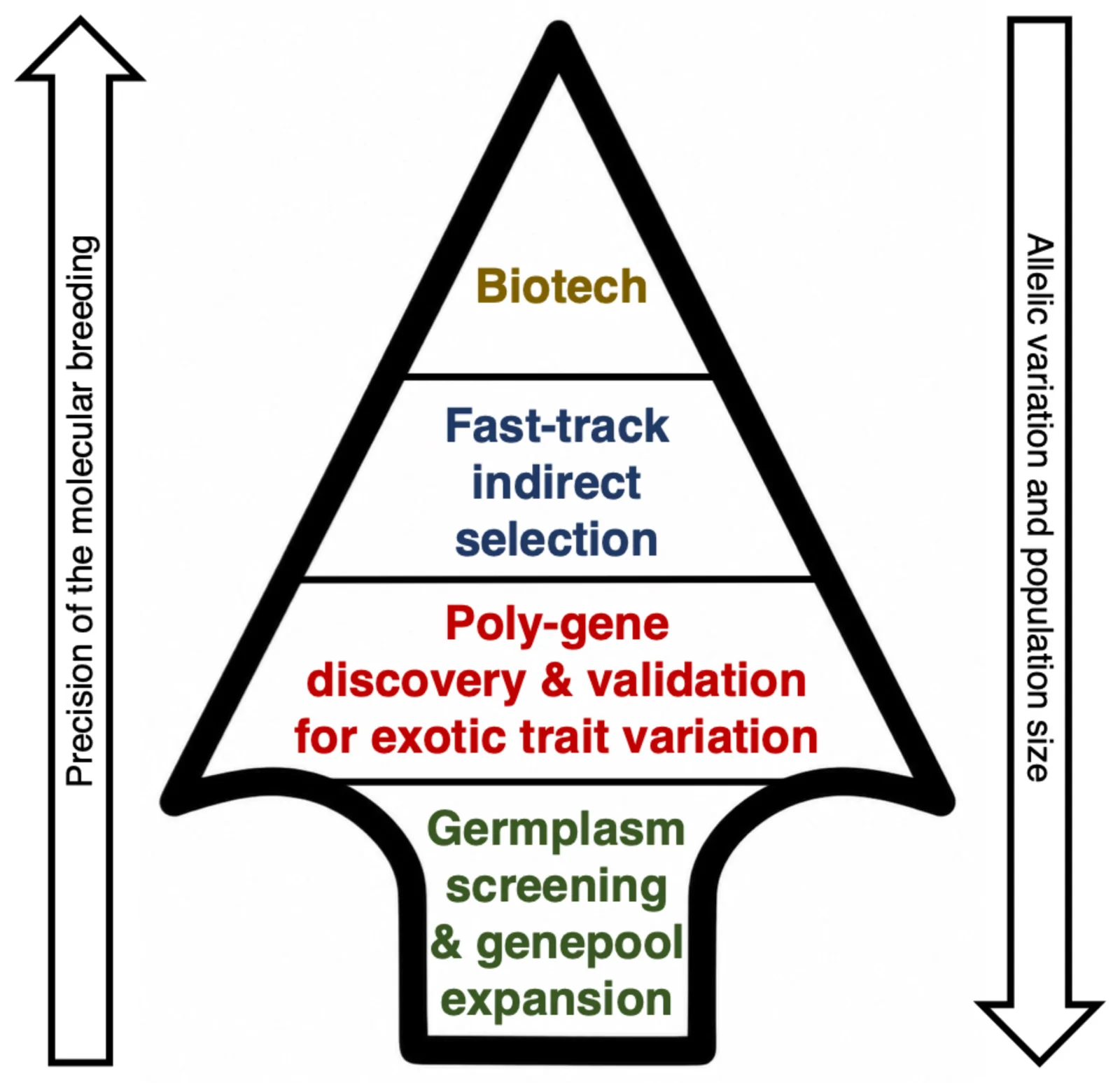
Conceptual framework illustrating in an arrow-like silhouette the progressive transition of modern plant breeding from germplasm exploration to precision gene editing-enhanced crop improvement. The arrow depicts a hierarchical continuum in which broad-scale germplasm screening and genepool expansion provide the foundational genetic diversity required for plant improvement. This diversity is subsequently translated into usable breeding resources through polygenic architecture discovery, candidate gene identification, and functional validation of exotic trait variation. The next stage involves the deployment of marker-assisted breeding, genomic prediction, and speed breeding to accelerate genetic gain by enabling early and more accurate selection. At the apex of the arrow, biotech—spanning transformation systems, synthetic biology, and genome editing—facilitates the direct manipulation and deployment of desirable alleles. The upward axis reflects the increasing precision of molecular breeding approaches, whereas the downward axis represents the progressive reduction in accessible allelic variation and effective population size as breeding advances from broad germplasm resources toward highly targeted genetic modifications.

**Figure 2:**
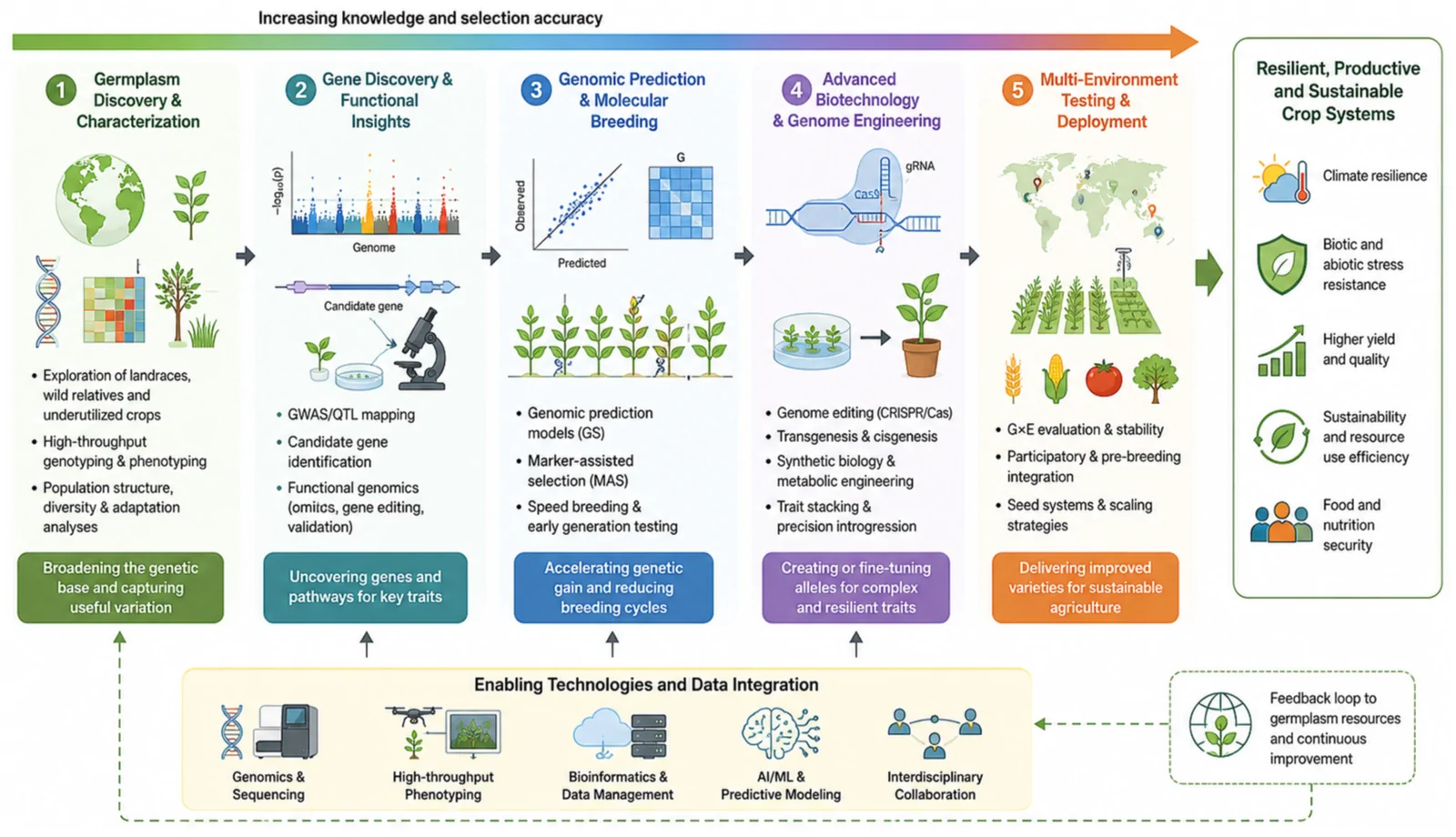
AI-inspired integrative framework for next-generation plant improvement as per the silhouette schematized in Figure 1 (inverted in this more detailed version). The workflow begins with germplasm discovery and characterization, where secondary genepools, landraces, wild relatives, underutilized species, and diverse breeding populations are surveyed using high-throughput genotyping and phenotyping approaches to capture adaptive variation. This diversity is subsequently translated into biological knowledge through candidate gene discovery and functional genomics, including genome-wide association studies (GWASs), quantitative trait locus (QTL) mapping, transcriptomics, and gene validation experiments that uncover the molecular basis of key agronomic traits. The resulting genomic information is then incorporated into genomic prediction and molecular breeding frameworks, enabling marker-assisted selection, genomic selection, and accelerated breeding cycles through early-generation prediction of breeding values. Advances in genome engineering, including transformation systems, synthetic biology, and CRISPR/Cas-based genome editing, further enable the targeted manipulation and deployment of beneficial alleles. Candidate genotypes are subsequently evaluated through multi-environment testing and deployment, where genotype-by-environment interactions, adaptation, and stability are assessed across diverse production scenarios before cultivar release. Together, these stages contribute to the development of productive, resilient and sustainable crop systems capable of enhancing yield potential, climate adaptation, biotic and abiotic stress resistance, nutritional quality and resource-use efficiency. The framework further highlights the enabling role of genomics, high-throughput phenotyping, bioinformatics, artificial intelligence (AI), machine learning (ML), genomic selection and interdisciplinary collaboration in supporting data-driven breeding decisions. Feedback loops between breeding outputs and germplasm resources further emphasize the inherent cyclical and cumulative nature of plant improvement, whereby newly generated knowledge and genetic resources continuously enrich future breeding efforts. Abbreviations are as follows: genome-wide association studies (GWASs), quantitative trait locus (QTL), genotype-by-environment interaction (G×E), marker-assisted selection (MAS), genomic selection (GS), clustered regularly interspaced short palindromic repeats (CRISPR), artificial intelligence (AI) and machine learning (ML).

**Table 1:** Summary of the compilation for the Special Issue “Plant Breeding and Genetics: New Findings and Perspectives” comprising a total of thirteen published studies. Abbreviations in the table are as follows: embryonal-suspensor masses (ESMs), genotyping-by-sequencing (GBS), Kompetitive Allele-Specific PCR (KASP), low-coverage whole-genome sequencing (lcWGS), new genomic techniques (NGTs), single-nucleotide polymorphism (SNP), specific-locus-amplified fragment sequencing (SLAF-seq), whole-genome resequencing (WGR). The table is sorted top-down by sub-discipline, approach, and species name. Full access to all contributions is provided in the Special Issue’s webpage, as declared in the Data Availability Statement at the end of the Editorial.

Species	Goal	Samples	Molecular Tools	Key Finding	Reference
Avocado (*Persea americana*)	Characterize with lcWGS germplasm from Tanzania	A total of 95 “plus trees” and lcWGS published data for 205 reference trees	Low-coverage whole-genome sequencing (lcWGS)	The Tanzanian genepool is mostly Mesoamerican- derived	Cortés et al. [32]
Eggplant (*Solanum melongena*)	Develop KASP markers for eggplant genotyping	A total of 286 eggplant accessions (resequencing data for 49)	Resequencing (224 SNPs) and KASP genotyping (96 SNPs)	KASP enables prioritizing 23 core SNPs and 56 core accessions	Wang et al. [40]
White clover (*Trifolium repens*)	Characterize the genetic and phenotypic variation in white clover	A total of 174 white clover accessions	SLAF-seq	Genetic and phenotypic clusters exhibit concordance	Shafiq et al. [44]
Crop species	Review genotyping technologies for plant germplasm characterization	Multi-species literature review	Literature review	Key tools include SNP arrays, GBS, DArTseq, RAD-seq, WGR, lcWGS	Werghi et al. [26]
*Bergenia* *purpurascens*	Functionally validate *bZIP* genes for heat tolerance at 45 °C	*B. purpurascens* and *Arabidopsis thaliana* (ecotype Columbia Col-0)	RNA-seq, RT-qPCR and heterologous validation	*BpbZIP37* enhances heat tolerance (seedling survival from 22.2% to 50%)	Zhu et al. [65]
Japanese larch (*Larix kaempferi*)	Functionally validate *LkmiR156s* in somatic embryogenesis	Immature zygotic embryos of *L. kaempferi* for ESMs	Heterologous validation in *A. thaliana*	*LkmiR156s* is coordinated with embryonic dormancy	Li et al. [66]
Desert poplar (*Populus euphratica*)	Characterize the *SnRK2* gene family for drought tolerance	*P. euphratica*’s genomic and transcriptomic resources	Transcriptomic data and subcellular localization	*PeSnRK2* genes are involved in stress response pathways	Jin et al. [67]
Salicaceae spp. and desert poplar (*P. euphratica*)	Characterize *ARF* genes for abiotic stress tolerance	*P. euphratica,* *Populus pruinosa* and *Salix sinopurpurea*	Transcriptomic data and qRT-PCR	*ARF* genes enable heterophylly as an adaptive mechanism	Song et al. [68]
Common bean (*Phaseolus vulgaris*) × Tepary Bean (*P. acutifolius*) hybrids	Forecast adaptation to the humid and dry regions at the Caribbean coast	A total of 87 advanced hybrid lines tested across three localities	A total of 15,645 GBS-derived SNPs coupled with genomic prediction	BayesC offers accurate model training for yield and biomass traits	López-Hernández et al. [80]
Common bean (*P. vulgaris*)	Synthesize molecular breeding strategies for fungal resistance	Eleven major fungal diseases	Literature review	Breeding fungal resistance urges due to climate change pressures	Benchimol-Reis et al. [84]
Sunflower (*Helianthus annuus*)	Characterize genotype-specific regeneration responses	A total of 18 sunflower genotypes	Contrasting regeneration media	Genotype-specific regeneration boosts gene editing and transgenesis	Ionas et al. [97]
Bananas (*Musa* spp.)	Bioengineer betalain-rich bananas using a RUBY system	*Musa acuminata* Cavendish banana cultivar	*Agrobacterium*- Mediated transformation	Screening time for betalain-rich bananas is reduced from 45 to 15 days	He et al. [98]
Crop species	Review NGTs in plant breeding	Multi-species literature review	CRISPR/Cas applications	NGTs face complex environmental and government issues	Koller [99]

## Data Availability

All thirteen contributing articles to the Special Issue “Plant Breeding and Genetics: New Findings and Perspectives” are accessible through https://www.mdpi.com/journal/ijms/special_issues/OY6V509K2A, as accessed on 7 September 2026.
